# Proteomic Analysis Provides Insights Into the Therapeutic Effect of GU-BEN-FANG-XIAO Decoction on a Persistent Asthmatic Mouse Model

**DOI:** 10.3389/fphar.2019.00441

**Published:** 2019-05-07

**Authors:** Li-wei Liu, Qiong-qiong Xing, Xia Zhao, Min Tan, Yuan Lu, Ying-mei Dong, Chen Dai, Yang Zhang

**Affiliations:** ^1^Affiliated Hospital of Nanjing University of Chinese Medicine, Nanjing, China; ^2^Jiangsu Key Laboratory of Pediatric Respiratory Disease, Institute of Pediatrics, Nanjing University of Chinese Medicine, Nanjing, China; ^3^Children’s Hospital of Soochow University, Suzhou, China; ^4^College of Life Sciences, Nanjing Agricultural University, Nanjing, China; ^5^Department of Chemistry and Institute of Biomedical Sciences of Shanghai Medical School, Fudan University, Shanghai, China

**Keywords:** GU-BEN-FANG-XIAO decoction, iTRAQ, chronic persistent asthma, macrophage, IPA

## Abstract

Gubenfangxiao decoction (GBFXD) is a traditional Chinese medicine based on a combination of Yu-Ping-Feng-San and Erchen decoctions. GBFXD has been widely used for decades in treating asthma at the Affiliated Hospital of Nanjing University of Chinese Medicine. Previously, GBFXD was found to reduce lung inflammation and airway remodeling; however, the underlying mechanism remains unknown. In this study, the effects of GBFXD on asthmatic mice were evaluated based on pathology and lung function; airway hyperresponsiveness (AHR) and pathology were compared among the two different mouse models utilized. Furthermore, the mechanism of action of GBFXD on asthmatic mice was analyzed using iTRAQ labeling technology combined with ingenuity pathway analysis (IPA). Modeling analysis of pre- and post-treatment proteins identified 75 differentially expressed proteins. These proteins were related to B-cell development, calcium-induced lymphocyte apoptosis, antigen presentation, and Th1 and Th2 activation pathways. Moreover, 68 differentially expressed proteins were identified in the GBFXD treatment group compared with the model group. Upstream regulatory factors predicted that interleukin (IL)-4 (necessary for inducing polarization of type 2 [M2] macrophages), cyclooxygenase, and prostaglandin E2 are significantly elevated in the model group. Based on IPA analysis, it was concluded that several pathways, including mitochondrial dysfunction and oxidative phosphorylation, are closely associated with the therapeutic effects of GBFXD in asthma. Moreover, the differential expression of several proteins, including the M2 markers, MRC1, ARG1, Retnla, Chil3, and CHIA, were validated by western blotting, confirming that GBFXD can reduce airway inflammation, which fits the pattern of an alternative M2 activation state, and attenuate AHR. Overall, our findings indicate that GBFXD significantly suppresses M2 macrophage polarization, providing further insights into the mechanism underlying the protective effects of GBFXD.

## Introduction

Asthma is one of the most common chronic, non-infectious airway diseases in children, with a global prevalence of 1–18% (Ebmeier et al., 2017; Papi et al., 2018). As a developing country, with the change of industrialization and lifestyle, the incidence of asthma in Chinese children is rising (Guo et al., 2018). Asthma is characterized by chronic airway inflammation, airway hyperresponsiveness and airway remodeling (Grainge et al., 2011). However, asthma in childhood and adult asthma is significantly different in epidemiology. Respiratory syncytial virus (RSV) is one of the main causes of recurrent asthma in children (Jartti and Gern, 2017; William et al., 2018). Moreover, epidemiological studies have shown that early infection with RSV can increase the risk of asthma in children (Carroll et al., 2017). Experiments have confirmed that the increased eosinophils can be found in airway of infants and mice after infection with RSV, which is associated with the involvement of viral prion-induced Th2 type cytokines as well as eosinophil chemotactic factors during RSV replication (Phipps et al., 2007). In addition, RSV infection can also cause damage to the airway surface (Zhang et al., 2002). At present, research on the role of RSV infection in asthma via proteomics has ever been yet reported.

The prevalence of asthma in children in China is 3.01% (Liu, 2019). However, the long-term use rate of ICS is only 57.8% (Liu and Chen, 2013). As a traditional medication, herbal compound has become a common alternative treatment for asthma in children in China and other Asian countries (Huang et al., 2013; Geng et al., 2016). At the molecular level, traditional Chinese medicine formulations are multi-targeted and multi-component. Combinatorial systems biology and omics technology currently provide powerful tools for addressing the complexities and mechanisms of traditional Chinese medicine formulations (Yue et al., 2017; Chun et al., 2018).

Gubenfangxiao decoction (GBFXD) has been used clinically for decades. It is consisted of traditional Chinese medicine compound Yupingfeng powder (YPF-P) and Ershen Tang (ECD). Previous clinical trials have confirmed the efficacy and safety for long-term prevention of asthma recurrence (Yuan et al., 2010). Subsequent studies have shown that GBFXD can reduce lung inflammation in OVA-sensitized mice, inhibit the expression of asthma susceptibility genes ORMDL3 and ADAM33, and reduce endoplasmic reticulum stress (ERS) (Huang et al., 2016; Lu et al., 2016). In this study, as a model of chronic persistant asthma, RSV-OVA-sensitized mice was established. iTRAQ-based proteomics studies and ingenuity pathway analysis (IPA) were performed to reveal the protein features of RSV-OVA-sensitized mice and investigate the underlying mechanisms of GBFXD.

**FIGURE 1:**
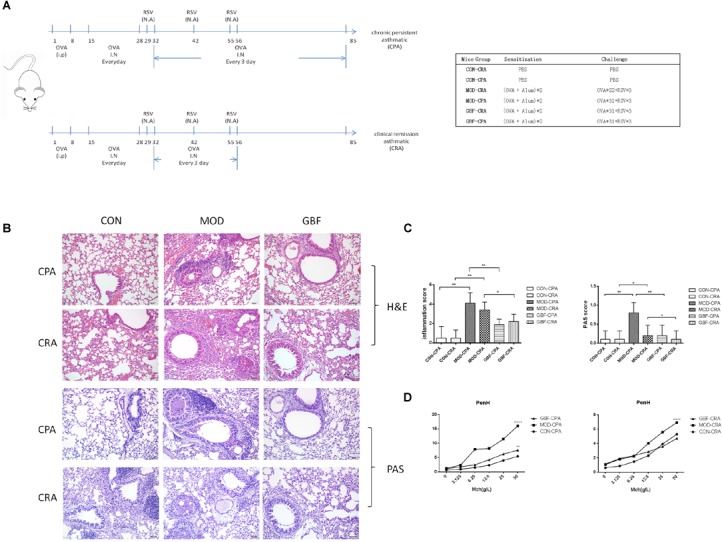
Effect of GBFXD treatment on airway hyperresponsiveness in ovalbumin-challenged mice and histological examination of lung tissue for airway inflammation (H&E and PAS staining). **(A)** Experimental scheme for the induction of airway inflammation in a mouse model. **(B)** H&E staining showing asthmatic inflammation. PAS staining identified epithelial goblet cells. **(C)** Total inflammation scores in all animal groups. The percentage of PAS-positive cells per bronchiole was calculated. **(D)** Airway responsiveness to aerosolized methacholine was measured with WBP. Mice were placed in the main chamber and nebulized first with PBS and then with increasing doses (3.125–50 mg/mL) of methacholine. Data represent the mean ± SEM of five independent experiments (two-way ANOVA by Tukey’s multiple comparisons test; ^∗∗^*p* < 0.01; ^∗∗∗^*p* < 0.0001; ^∗∗∗∗^*p* < 0.0001).

## Materials and Methods

### Animal Experiments and Drug Treatments

#### GBFXD Preparation

GBFXD, which consists of 11 components (Table 1), was purchased from the Jiangsu Province Hospital of Chinese Medicine (Jiangsu, China) and authenticated by Dr. Sheng-Jin Liu of the College of Pharmacy, Nanjing University of Chinese Medicine, Nanjing, China. The principal components of GBFXD were prepared and identified by high-performance liquid chromatography-ultraviolet in a previous study (Lu et al., 2016).

**Table 1:** GBFXD composition.

Component	Part	Weight (g)
Radix astragali preparata cum melle (Zhi Huang Qi)	Root	15
*Codonopsis* radix (Dang Shen)	Root	10
*Atractylodis macrocephalae* rhizoma (Bai Zhu)	Rhizome	10
Poria (Fu Ling)	Sclerotia	10
Calcined concha Ostreae (Duan Mu Li)	Shell	15
*Periostracum cicadae* (Chan Tui)	Slough	6
Citri pericarpium reticulatae (Chen Pi)	Peel	6
*Saposhnikoviae* radix (Fang Feng)	Root	3
Flos Magnoliae (Xin Yi)	Bud	6
*Schisandrae Chinensis* fructus (Wu Wei Zi)	Fruit	6
*Glycyrrhizae* radix et rhizoma (Zhi Gan Cao)	Rhizome and root	3

#### Animal Model Establishment

Four-week-old, female, specific-pathogen-free BALB/c mice (16–18 g) were purchased from Suzhou JOINN Laboratories Co., Ltd. (Jiangsu, China). The animals were housed in the experimental animal center of Nanjing University of Chinese Medicine and maintained under a 12 h light/dark cycle at a constant temperature of 22 ± 2°C. Cages, bedding, food, and water were sterilized before use. Mice were sensitized with 20 μg intraperitoneal OVA injections (grade II; Sigma-Aldrich, St. Louis, MO, United States), after which the clinical remission asthmatic (CRA) and chronic persistent asthmatic (CPA) models were established at two different challenge frequencies. The excitation models included 2.5% OVA atomization and RSV in nasal drop form with a titer of 1.0 × 10 TCID_50_/mL (Figure 1A). The mice were randomly divided into six groups as follows, CON-CRA control group, MOD-CRA model group, GBF-CRA (36 g/kg/d) treatment group, CON-CPA control group, MOD-CPA model group, and GBF-CPA (36 g/kg/d) treatment group. Prior to the experiments, there were no significant differences among the groups in terms of animal weight. All experimental procedures were performed in accordance with the National Institutes of Health Guidelines for Laboratory Animals and approved by the Animal Ethics Committee of Nanjing University of Chinese Medicine [no. SYXK (Su) 2014–0001].

### Proteomics

#### Protein Extraction and Digestion

Lungs were excised, immediately frozen at −80°C, and ground in liquid N_2_. Cold RIPA extraction buffer (Beyotime, Haimen, China) was added to the pulverized tissue and then the mixture was sonicated. Next, 1 mM phenylmethanesulfonyl fluoride (Beyotime), 2 mM ethylenediaminetetraacetic acid, 10 mM dithiothreitol, and protease inhibitor cocktails (Roche, Basel, Switzerland) were added, after which the mixture was centrifuged at 4°C and 30,000 × *g* for 15 min. The supernatant was collected and added to five volumes of cold acetone containing 10% (v/v) trichloroacetic acid, thoroughly mixed, and incubated at −20°C overnight. The mixture was centrifuged again at 4°C and 30,000 × *g* and the supernatant was discarded. The precipitate was then washed three times with chilled acetone, dissolved in RIPA buffer, and air-dried. Proteins were quantified with a BCA kit (Thermo Fisher Scientific, Waltham, MA, United States), after which 300 μg of total protein was mixed with sequencing-grade trypsin (Promega, Madison, WI, United States) at an enzyme-to-protein ratio of 1:50 and incubated at 37°C for 16 h. Peptides obtained from the digestion were dried by vacuum centrifugation.

#### iTRAQ Labeling and High-pH Reverse-Phase (RP) Fractionation

Peptides were processed using 4-plex iTRAQ reagent (AB Sciex, Framingham, MA, United States) according to manufacturer’s instructions. Control samples were labeled with 116 iTRAQ tags, model samples were labeled with 115 iTRAQ tags, GBFXD samples were labeled with 114 iTRAQ tags, and the mixtures were labeled with 117 iTRAQ tags. High pH RP fractionation was then performed with the U3000 HPLC chromatography system (Thermo Fisher Scientific). The iTRAQ-labeled peptide mixtures were reconstituted with 100 μL of high pH RP buffer A (98% H_2_O, 2% acetonitrile; pH 10.0) and loaded onto a C18 column with a particle size of 1.7 μm (2.1 mm × 100 mm; Waters Corporation, Milford, MA, United States). The column was eluted with the following gradient program, 3–18% buffer B (2% H_2_O, 98% acetonitrile; pH 10.0) for 30 min; 18–32% B for 15 min; 32–98% B for 6 min; and holding at 98% B for 15 min. The flow rate was 0.2 mL/min and elution was monitored by measuring the absorbance at 214 nm.

#### Liquid Chromatography-Tandem Mass Spectrometry (LC-MS/MS) Analysis

Peptides were re-dissolved in buffer A (2% acetonitrile, 0.1% formate) and centrifuged at 4°C and 20,000 × *g* for 10 min. The final peptide concentration of each fraction was ∼0.2 μg/μL. The peptides (10 μL) were then loaded onto a 2-cm C18 trap column using the Nano LC System autosampler (Thermo Fisher Scientific) and eluted onto a 15-cm analytical C18 column with an inner diameter of 75 μm using a 3–55% buffer B (84% acetonitrile, 0.1% formate) gradient. The elution process ran for 112 min at a flow rate of 300 nL/min. The peptides were ionized via nano-electrospray ionization at a voltage of 2.2 kV. Data-dependent MS/MS was performed using an LTQ-Orbitrap XL mass spectrometer (Thermo Fisher Scientific). Using the MS1 full scan at a resolution of 60,000, the five most abundant precursor ions surpassing the 5,000 count threshold were selected for MS/MS analysis at a dynamic exclusion duration of 60 s. Normalized collision energy for high-energy collision dissociation (HCD) was set to 40.0. Finally, the ion fragments were detected in the Orbitrap at a resolution of 7,500 m/z scan ranges were 350–1800 and 100–1800 Da for the MS1 and the MS2 scans, respectively.

#### Protein Identification and Quantification

Raw MS data were converted into.MGF files using Proteome Discoverer v.1.4 (Thermo Fisher Scientific). Proteins were identified with the SEQUEST search engine and considered differentially expressed when their fold changes were > 1.5 or <0.66 and *p* < 0.05. Parameters required for qualification are listed in Table 2.

**Table 2:** Parameters required for qualification.

Parameter
Enzyme: trypsin
Miss cleavage: 2
MS/MS tolerance ppm: 10
Fixed modification: carbamidomethylation (C)
Variable modification: oxidation (M) and deamidation (NQ)
Unique peptide: 2
Peptide FDR: 0.01

#### Bioinformatic Analysis of Differentially Expressed Proteins (DEPs)

Protein-encoding genes were functionally categorized according to biological process, molecular function, and cellular components by the PANTHER gene classification system. Proteomic data were analyzed by Ingenuity^®^ Pathways Analysis (IPA; Qiagen, Hilden, Germany) to elucidate the hidden biological significance of the experimental data. Biological information obtained from data analysis is presented as biological function/disease, canonical pathways, networks, and upstream regulators.

### Histopathology

Lung tissues were infused with 4% paraformaldehyde, embedded in paraffin, and processed for histology. Paraffin-embedded samples were sectioned (5-mm thick) and stained with hematoxylin and eosin (H&E) to examine the extent of peribronchial inflammation. Glandular hyperplasia was analyzed in randomly selected samples using the periodic acid Schiff (PAS) staining method.

### Airway Hyperresponsiveness (AHR) Testing

BABL/c mice in their natural state were placed in Whole Body Plethysmography(WBP) and allowed to stabilize for 3 min to adapt to the environment. Basic physiological respiratory parameters were measured for 3 min, after which normal saline was added to the inhalation tubes in the boxes. Diluted methacholine (300 μL) was then injected into the boxes at a concentration of 0, 3.125, 6.25, 12.5, and 25 g/L. The mist generation rate was adjusted to 50%. For every minute of nebulization and inhalation, Enhanced pause (Penh) values were calculated using the software of the associated computer interface; the average Penh value at each challenge dose was calculated 3 min after inhalation.

### Validation

Total RNA from the lung samples was isolated using TRIzol reagent (Takara Bio, Kusatsu, Japan) according to manufacturer’s instructions. Reverse transcription master mix (G490) and EvaGreen qPCR master mix (MasterMix-ER) were purchased from Applied Biological Materials Inc. (Richmond, BC, Canada). *ARG1, MRC1, Retnla, CHIL3*, *TNF*, and *IFNG* mRNA levels were determined by quantitative reverse transcription (qRT)-PCR using GAPDH as a reference gene (Table 3). Expression levels of ARG1, CHIA, and Retnla (Abcam, Cambridge, United Kingdom) in lung tissue were also measured by western blotting using β-actin as loading control. Protein samples from the lungs were fractionated via SDS-PAGE. Three independent experiments were performed.

**Table 3:** Primer sequences.

Gene	Forward primer	Reverse primer	Expected product size (bp)
*Arg1*	CCGAGGATGGAGAGCAGCTAGG	CCTGAGAGTCTGTGCCAATGAGC	150
*Retnla*	ACTTCTTGCCAATCCAGCTAACTATCC	GCAGTGGTCCAGTCAACGAGTAAG	204
*CHIL3*	CAGTGTTCTGGTGAAGGAAATG	ACCCAGACTTGATTACGTCAAT	119
*Mrc1*	GTCTGAGTGTACGCAGTGGTTGG	TCTGATGATGGACTTCCTGGTAGCC	85
*IFNG*	GCGTCATTGAATCACACCTG	TGAGCTCATTGAATGCTTGG	129
*TNF*	GCGACGTGGAACTGGCAGAAG	CATCGGCTGGCACCACTAGTTG	327
*GAPDH*	CGTGTTCCTACCCCCAATGT	TGTCATACTTGGCAGGTTT	104

### Data Analysis

Data are expressed as the mean ± standard error of the mean (SEM). All statistical analyses were performed using GraphPad Prism 6.0 (GraphPad Software Inc., San Diego, CA, United States). Statistical analysis was performed using one-way analysis of variance (ANOVA) followed by Dunnett’s *post hoc* test to determine the statistical significance. Differences were considered significant when *p* < 0.05.

## Results

### GBFXD Alleviates Airway Inflammation and AHR Caused by Persistent Challenge

We improved the previous method (Lu et al., 2016) and established a CPA model that more closely simulates chronic persistent asthmatic characteristics than the one previously tested. H&E staining, PAS staining, and AHR of the two models tested were compared. In both models, the mice developed typical inflammatory changes including bronchial congestion and inflammatory cell infiltration. Mucus expression in the airways was evaluated by quantifying and comparing PAS-positive cells between the two models; the CPA model presented with more prominent goblet cell hyperplasia and mucus overproduction. After GBFXD treatment, however, the average number of PAS-stained goblet cells was greatly reduced (Figure 1B,C). Asthma is an airway obstruction often accompanied by varying degrees of AHR. As the Penh metric can reflect airway responsiveness and Penh values are accepted AHR indicators, we calculated Penh values after induction with a methacholine concentration gradient. We found higher AHR in the CPA model than in the CRA model, with GBFXD showing significantly reduced AHR (Figure 1D).

### Proteomic Expression Patterns

**FIGURE 2:**
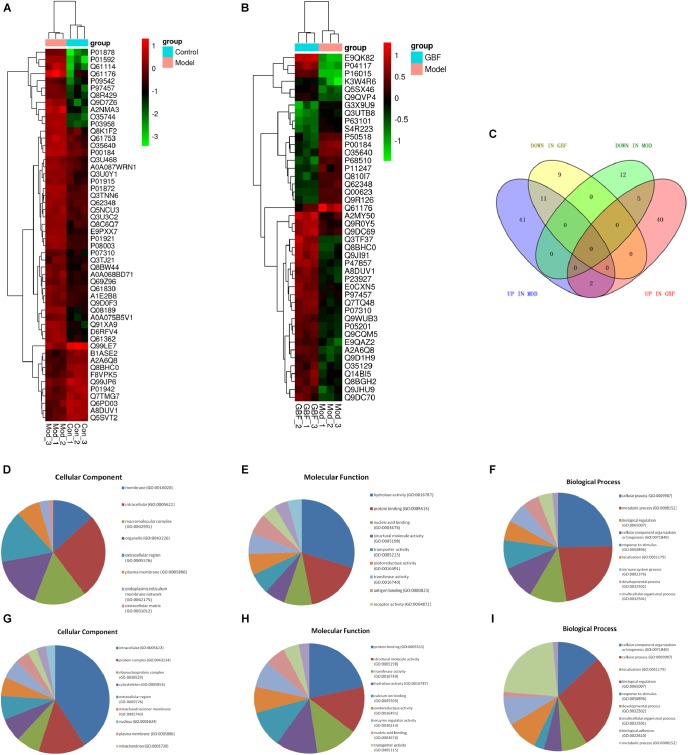
Protein expression profiles. **(A,B)** Heatmap of the expression levels of differentially expressed proteins (DEPs) compared with those of the model group quantified by iTRAQ-based stable isotope tagging. **(C)** Venn diagrams showing the distribution of shared DEPs. Blue represents upregulated proteins in the model group (up in MOD), green represents downregulated proteins in the model group (down in MOD), pink represents upregulated proteins in the GBFXD group (up in GBF), and yellow represents downregulated proteins in the GBFXD group (down in GBF). Overlapping sections indicate the number of common proteins across different groups. GO, gene ontology enrichment analysis. GO analysis of DEPs after modeling; **(D)** biological process, **(E)** cellular component, and **(F)** molecular function. GO analysis of DEPs after GBFXD treatment; **(G)** biological process, **(H)** cellular component, and **(I)** molecular function.

To compare the pathological and pulmonary function results, we selected the CPA model for further proteomic experiments. A total of 1,956 proteins were identified by iTRAQ with a < 1% false discovery rate (FDR) after analyzing three biological replicates of mouse lung tissue. Proteins with significantly altered expression levels at a fold change cut-off of > 1.5 relative to the model group were considered DEPs (*p* < 0.05). We found 71 DEPs in the model group compared with control; 17 proteins were downregulated while 54 proteins were upregulated (Figure 2A and Table 4). Moreover, a total of 68 proteins were differentially expressed after GBFXD treatment compared with that of the model group; 47 proteins were upregulated and 20 were downregulated (Figure 2B and Table 5). EIF4B, HBA1/HBA2, Hist1h1e, HLA-A, and LYVE1 were repressed in the model group but were found upregulated after GBFXD treatment. In contrast, ANXA8/ANXA8L1, APOA, ARG1, CHIA, CYP1A1, FTL, H2AFZ, PLPP3, Retnla, S100A4, and TSN were induced in the model group but downregulated in response to GBFXD treatment (Figure 2C). Furthermore, DEPs were classified using the PANTHER gene classification systems and the enriched gene ontology (GO) terms were found enriched compared with the control group, hydrolase activity (GO:0016787), protein binding (GO:0005515), metabolic process (GO:0008152), and response to stimuli (GO:0050896). DEPs between the GBFXD and model groups were mainly involved in metabolic processes (GO:0008152), cellular processes (GO:0009987), cellular component organization or biogenesis (GO:0071840), intracellular processes (GO:0005622), and protein binding (GO:0005515; Figure 2D–I).

**Table 4:** Differentially expressed proteins (DEPs) in the CON group compared with the MOD group.

Expression fold change	ID	Symbol	Entrez gene name
−4.011	O88990	Actn3	Actinin alpha 3
−4.834	P03958	ADA	Adenosine deaminase
−1.877	O35640	ANXA8/ANXA8L1	Annexin A8 like 1
−1.563	Q810I7	APOA4	Apolipoprotein A4
−3.331	Q61176	ARG1	Arginase 1
−1.622	Q69Z96	ATP13A1	ATPase 13A1
−2.623	Q8R429	ATP2A1	ATPase sarcoplasmic/endoplasmic reticulum Ca^2+^ transporting 1
1.675	B1ASE2	ATP5H	ATP synthase, H+ transporting, mitochondrial Fo complex subunit D
−6.174	Q61114	BPIFB1	BPI fold containing family B member 1
−1.556	Q3TNN6	CAPG	Capping actin protein, gelsolin like
−2.274	Q61362	CHI3L1	Chitinase 3 like 1
−2.707	Q91XA9	CHIA	Chitinase, acidic
−6.08	O35744	Chil3/Chil4	Chitinase-like 3
−1.812	P07310	CKM	Creatine kinase, M-type
−4.014	Q9D7Z6	CLCA1	Chloride channel accessory 1
1.825	P02463	COL4A1	Collagen type IV alpha 1 chain
−1.871	D3Z315	COPE	Coatomer protein complex subunit epsilon
−1.82	P00184	CYP1A1	Cytochrome P450 family 1 subfamily A member 1
1.636	Q922K6	EIF4B	Eukaryotic translation initiation factor 4B
−2.16	Q3THE6	FTL	Ferritin light chain
−1.552	Q3UA95	H2AFZ	H2A histone family member Z
1.609	P01942	HBA1/HBA2	Hemoglobin subunit alpha 2
1.753	A8DUV1	HBA1/HBA2	Hemoglobin subunit alpha 2
2.24	P43274	Hist1h1e	Histone cluster 1, H1e
1.528	P01900	HLA-A	Major histocompatibility complex, class I, A
−1.82	A0A068BD71	HLA-DQA1	Major histocompatibility complex, class II, DQ alpha 1
−1.809	P01921	HLA-DQB1	Major histocompatibility complex, class II, DQ beta 1
−1.754	P01915	HLA-DRB5	Major histocompatibility complex, class II, DR beta 5
−1.594	Q861Q5	HM13	Histocompatibility minor 13
−1.791	Q66K04	Igh	Immunoglobulin heavy chain complex
−1.885	P01868	IGHG1	Immunoglobulin heavy constant gamma 1 (G1m marker)
−1.905	P01872	IGHM	Immunoglobulin heavy constant mu
−1.94	A0A075B5V1	Ighv1-31	Immunoglobulin heavy variable 1–31
−2.789	A0A075B5J9	Igkv17-127	Immunoglobulin kappa variable 17–127
−2.286	A0A0B4J1J0	Igkv4-50	Immunoglobulin kappa variable 4–50
−5.083	Q9U410	Igkv4-55	Immunoglobulin kappa variable 4–55
−3.655	A0A0B4J1J2	Igkv5-43	Immunoglobulin kappa chain variable 5–43
−7.651	P01592	JCHAIN	Joining chain of multimeric IgA and IgM
1.668	Q8BHC0	LYVE1	Lymphatic vessel endothelial hyaluronan receptor 1
−1.705	Q6ZQJ2	METAP1	Methionyl aminopeptidase 1
−1.568	Q61830	MRC1	Mannose receptor C-type 1
−2.894	P97457	MYLPF	Myosin light chain, phosphorylatable, fast skeletal muscle
1.555	Q3TF41	NAP1L1	Nucleosome assembly protein 1 like 1
1.534	Q7TMG7	NPR3	Natriuretic peptide receptor 3
−2.226	Q62422	OSTF1	Osteoclast stimulating factor 1
−1.722	P08003	PDIA4	Protein disulfide isomerase family A member 4
1.556	D3Z375	PEA15	Phosphoprotein enriched in astrocytes 15
−1.885	Q61753	PHGDH	Phosphoglycerate dehydrogenase
−1.921	O35405	PLD3	Phospholipase D family member 3
−1.741	Q99JY8	PLPP3	Phospholipid phosphatase 3
1.649	Q6PD03	PPP2R5A	Protein phosphatase 2 regulatory subunit B’alpha
−1.637	Q8C6Q7	PTPRC	Protein tyrosine phosphatase, receptor type C
2.09	Q99LE7	PXN	Paxillin
1.673	Q9Z0J1	RECK	Reversion inducing cysteine rich protein with kazal motifs
−5.949	Q9JM62	REEP6	Receptor accessory protein 6
−2.315	Q9EP95	Retnla	Resistin-like alpha
−2.726	P07091	S100A4	S100 calcium binding protein A4
−3.757	Q6ZPE2	SBF1	SET binding factor 1
−2.48	D6RFV4	SEC11C	SEC11 homolog C, signal peptidase complex subunit
−2.004	Q3TT70	SEL1L	SEL1L ERAD E3 ligase adaptor subunit
1.583	P07759	SERPINA3	Serpin family A member 3
−2.036	Q3U0Y1	Serpina3g (includes others)	Serine (or cysteine) peptidase inhibitor, clade A, member 3G
−2.852	G3X9V8	SERPINB4	Serpin family B member 4
−5.387	P97298	SERPINF1	Serpin family F member 1
−2.29	Q08189	TGM3	Transglutaminase 3
−4.72	A2AQJ7	TMEM87A	Transmembrane protein 87A
1.547	Q5SVT2	TRIM16	Tripartite motif containing 16
−1.52	Q62348	TSN	Translin
1.565	Q9CQU0	TXNDC12	Thioredoxin domain containing 12
−1.979	E9PXX7	TXNDC5	Thioredoxin domain containing 5
−1.803	Q0VGU5	VKORC1L1	Vitamin K epoxide reductase complex subunit 1 like 1

**Table 5:** DEPs in the GBF group compared with the MOD group.

Expression fold change	ID	Symbol	Entrez gene name
−1.548	O89054	ACTB	Actin beta
1.871	Q9JI91	ACTN2	Actinin alpha 2
1.698	Q9R0Y5	AK1	Adenylate kinase 1
−1.711	O35640	ANXA8/ANX A8L1	Annexin A8 like 1
−1.784	Q00623	APOA1	Apolipoprotein A1
−1.642	Q810I7	APOA4	Apolipoprotein A4
−1.633	Q61176	ARG1	Arginase 1
1.554	Q3TJD4	ATP5F1	ATP synthase, H+ transporting, mitochondrial Fo complex subunit B1
−1.99	O54962	BANF1	Barrier to autointegration factor 1
2.894	P16015	CA3	Carbonic anhydrase 3
−1.533	Q91XA9	CHIA	Chitinase, acidic
1.626	P07310	CKM	Creatine kinase, M-type
1.76	P23927	CRYAB	Crystallin alpha B
−2.023	P00184	CYP1A1	Cytochrome P450 family 1 subfamily A member 1
−1.781	Q9R126	Ear7	Eosinophil-associated, ribonuclease A family, member 7
1.613	Q922K6	EIF4B	Eukaryotic translation initiation factor 4B
3.173	P04117	FABP4	Fatty acid binding protein 4
−1.7	G3X9U9	FIS1	Fission, mitochondrial 1
−1.743	Q3THE6	FTL	Ferritin light chain
1.504	Q3UDC0	GBP2	Guanylate binding protein 2
1.742	E9QAZ2	Gm10020	Ribosomal protein L15 pseudogene
1.59	D3YVC6	Gm21596/Hmgb1	High mobility group box 1
1.655	P05201	GOT1	Glutamic-oxaloacetic transaminase 1
1.758	E0CXN5	GPD1	Glycerol-3-phosphate dehydrogenase 1
−1.56	Q3UTB8	GRPEL1	GrpE like 1, mitochondrial
−1.636	Q3UA95	H2AFZ	H2A histone family member Z
1.792	Q61425	HADH	Hydroxyacyl-CoA dehydrogenase
1.727	A8DUV1	HBA1/HBA2	Hemoglobin subunit alpha 2
2.253	P43274	Hist1h1e	Histone cluster 1, H1e
1.592	P01900	HLA-A	Major histocompatibility complex, class I, A
1.676	Q9JHU9	ISYNA1	Inositol-3-phosphate synthase 1
2.221	Q8BHC0	LYVE1	Lymphatic vessel endothelial hyaluronan receptor 1
1.58	F6TBV1	MAGT1	Magnesium transporter 1
1.63	E0CYU5	MAT2B	Methionine adenosyltransferase 2B
1.541	Q9D1H9	MFAP4	Microfibrillar associated protein 4
2.021	P34884	MIF	Macrophage migration inhibitory factor (glycosylation-inhibiting factor)
4.167	E9QK82	MPZ	Myelin protein zero
2.178	Q3TF37	MYBPC3	Myosin binding protein C, cardiac
1.517	Q02566	MYH6	Myosin heavy chain 6
1.782	A2A6Q8	MYL4	Myosin light chain 4
1.661	Q9QVP4	MYL7	Myosin light chain 7
1.609	P97457	MYLPF	Myosin light chain, phosphorylatable, fast skeletal muscle
1.591	Q14BI5	MYOM2	Myomesin 2
1.825	O35683	NDUFA1	NADH:ubiquinone oxidoreductase subunit A1
1.509	Q9DC69	NDUFA9	NADH:ubiquinone oxidoreductase subunit A9
1.545	Q9DC70	NDUFS7	NADH:ubiquinone oxidoreductase core subunit S7
1.829	Q3TF69	PCBP2	Poly(rC) binding protein 2
1.631	P47857	PFKM	Phosphofructokinase, muscle
1.634	O35129	PHB2	Prohibitin 2
1.513	Q3TD51	PICALM	Phosphatidylinositol binding clathrin assembly protein
−1.698	Q99JY8	PLPP3	Phospholipid phosphatase 3
1.53	Q9WUB3	PYGM	Glycogen phosphorylase, muscle associated
−1.572	Q9EP95	Retnla	Resistin like alpha
1.523	Q9D1R9	Rpl34 (includes others)	Ribosomal protein L34
1.88	Q9JJI8	RPL38	Ribosomal protein L38
1.666	P62267	RPS23	Ribosomal protein S23
−1.779	Q9JL08	S100A1	S100 calcium binding protein A1
−1.59	P07091	S100A4	S100 calcium binding protein A4
1.534	Q8BGH2	SAMM50	SAMM50 sorting and assembly machinery component
1.701	P36536	SAR1A	Secretion associated Ras related GTPase 1A
−1.541	A2BE92	SET	SET nuclear proto-oncogene
1.804	Q7TQ48	SRL	Sarcalumenin
1.924	K3W4R6	TNNT2	Troponin T2, cardiac type
−1.6	Q62348	TSN	Translin
1.595	Q9CQM5	TXNDC17	Thioredoxin domain containing 17
1.93	Q78IK2	USMG5	Up-regulated during skeletal muscle growth 5 homolog (mouse)
−1.707	P68510	YWHAH	Tyrosine 3-monooxygenase/tryptophan 5-monooxygenase activation protein eta

### Analysis of Proteomic Data via IPA

#### Proteomic Analysis of the Model

**FIGURE 3:**
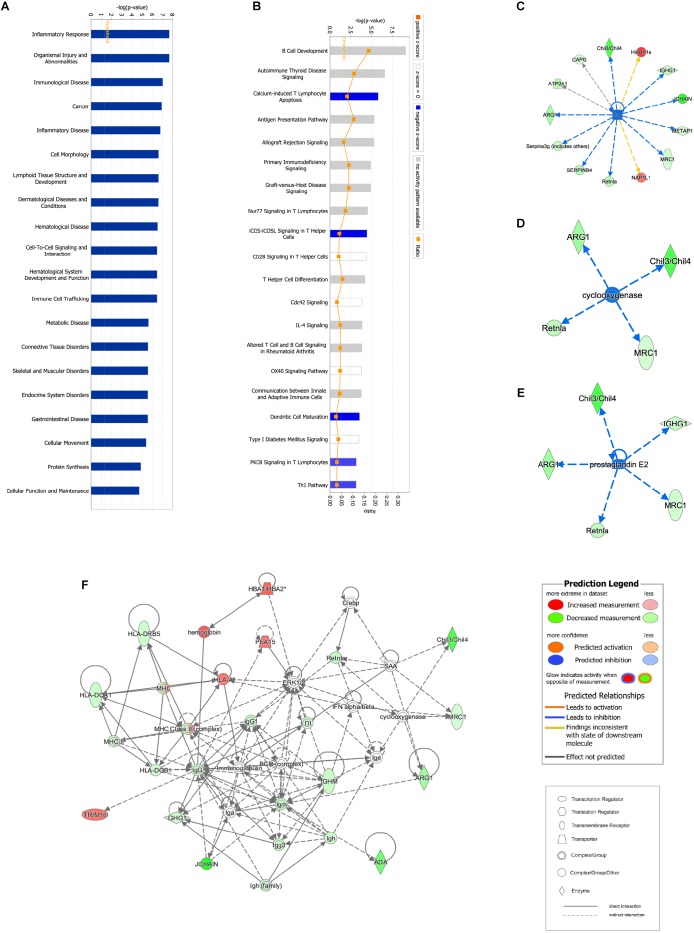
Bioinformatic analysis of DEPs between the control and model groups using Ingenuity Pathway Analysis (IPA) and *z*-score algorithms. **(A)** Biological function enrichment. **(B)** Canonical pathways enrichment (*p*-values for all functions and pathways presented are < 0.05). **(C–E)** Upstream regulatory factors predicted based on DEPs. **(F)** Relationships between DEPs and proteins predicted to be involved in interactions, as well as relevant signaling pathways, are presented. Statistical predictions of the networks were based on the Ingenuity Knowledge base. Red nodes represent upregulated proteins and green nodes represent downregulated proteins.

Proteomic data were analyzed via IPA, in which proteins are analyzed as a network using canonical pathways, disease networks, and predicted upstream regulators. Bioinformatic analysis showed that the model was involved in 165 pathways and 80 biological functions (Figure 3). Functional enrichments in inflammatory response, respiratory diseases, and inflammatory disease suggest that this model exhibits significant pulmonary inflammation. The high degree of enrichment in organismal injury and abnormalities, connective tissue disorders, and tissue morphology also suggests substantial changes in histomorphology. Humoral immune response and immune cell trafficking were also highly involved. The top 20 enriched disease and function terms are shown in Figure 3A. Canonical pathway analysis of the data revealed that our established model significantly activated multiple pathways in the mouse immune system; these included B-cell development, calcium-induced lymphocyte apoptosis, antigen presentation, iCOS-iCOSL signaling in T-helper cells, dendritic cell maturation, PKCθ signaling in T-lymphocytes, and Th1 and Th2 activation pathways. We also found that the DEPs were associated with 30 metabolic pathways. Upstream regulator predictions suggested that six inflammatory cytokines were upregulated and one downregulated (*Z*-Score > 1 or <−1); among the upregulated cytokines, the classical Th2 cytokines interleukin (IL)-4 and IL-5 drive M2 macrophage polarization in asthma pathogenesis (Table 6). Moreover, ARG1, MRC1, Chil3/Chil4, CHIA, and Retnla are typical cytokines secreted by M2 macrophages typical M2 cytokines that were found among the DEPs. In addition, GSF1, GSF2, cyclooxygenase, and prostaglandin E2 were significantly predicted upstream. These findings suggest that macrophages play an important role in this asthma model (Table 6). We further edited and expanded the functional network according to the molecular relationship most strongly correlated with the DEPs. The results showed that the DEPs constitute a reliable network and participate in inflammation and immune cell trafficking. The expression of the M2-type macrophage factors ARG1, MRC1, Chil3/Chil4, and Retnla is related to serum amyloid A (SAA) and cyclooxygenase. HBA1/HBA2 is a common biochemical indicator in the network. Therefore, there is abnormal blood oxygen saturation in the reaction state (Figure 3F).

**Table 6:** Upstream analysis of CON vs. the MOD group.

Upstream regulator	Predicted activation state	Activation *z*-score	*p*-value of overlap	Target molecules in dataset
Cyclooxygenase	Inhibited	−2	2.86E-07	ARG1, Chil3/Chil4, MRC1, Retnla
IL4	Inhibited	−2.412	1.63E-06	ARG1, ATP2A1, CAPG, Chil3/Chil4, Hist1h1e, IGHG1, JCHAIN, METAP1, MRC1, NAP1L1, Retnla, Serpina3g (includes others), SERPINB4
IL21	Inhibited	−2.236	1.88E-04	ARG1, HLA-DRB5, IGHG1, PTPRC, Serpina3g (includes others)
IL5	Inhibited	−2.376	2.05E-04	IGHG1, IGHM, JCHAIN, PTPRC, S100A4, Serpina3g (includes others)
Prostaglandin E2	Inhibited	−2.191	2.23E-03	ARG1, Chil3/Chil4, IGHG1, MRC1, Retnla
CSF1	Inhibited	−2	2.54E-03	ARG1, MRC1, PTPRC, Retnla
CSF2	Inhibited	−2.219	1.22E-02	ADA, ARG1, HLA-DQB1, MRC1, PHGDH

#### Proteomic Analysis Revealed the Therapeutic Effects of GBFXD

**FIGURE 4:**
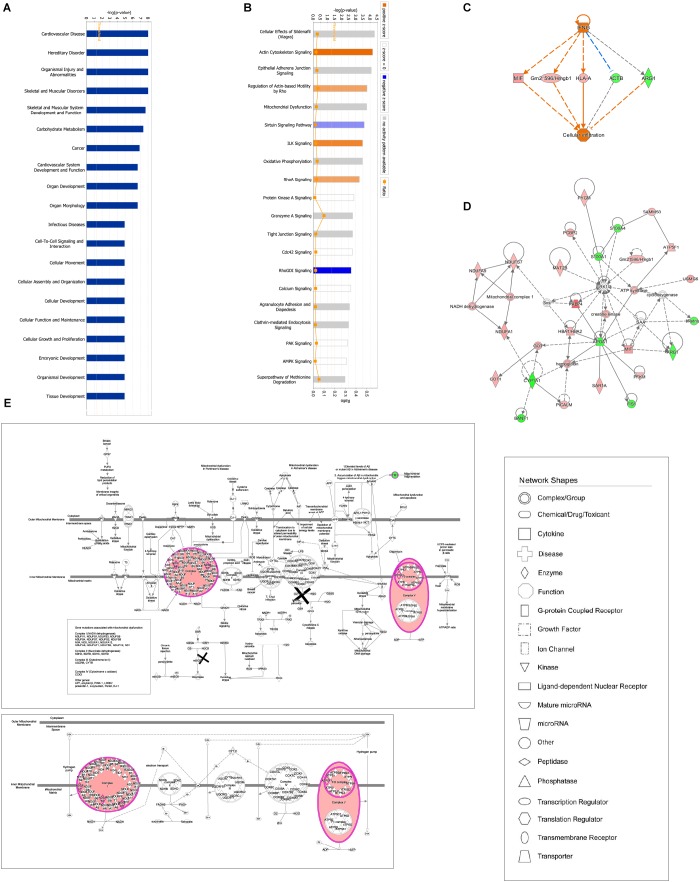
Bioinformatic analysis of DEPs in the lung after GBFXD treatment. **(A)** Biological function enrichment. **(B)** The most enriched pathways as a summary of DEPs between the GBFXD and model groups. **(C)** Regulatory effects resulting from integrating target molecules, upstream factors, and downstream biological functions. **(D)** The most credible interaction networks. **(E)** IPA of mitochondrial function and the oxidative phosphorylation pathway.

Ingenuity pathway analysis correlation analysis was performed to compare the GBFXD treatment and model groups and a total of 159 pathways were found to be involved. Pathways with significant regulation included actin cytoskeleton signaling, rho regulation of actin-based motility, ILK signaling, and sirtuin signaling. In addition, highly enriched pathways included mitochondrial dysfunction and oxidative phosphorylation. DEPs were found associated with 76 biological programs, 6 metabolic pathways, the immune program, and morphologically related programs; lipid and carbohydrate metabolism were the most enriched. Networks constructed with DEPs suggested that GBFXD treatment may have increased mitochondrial respiratory function and oxidative phosphorylation (Figure 4). Furthermore, we compared the highest scoring internetworks predicted by the two protein groups. Seven points were coincident (ARG1, cyclooxygenase, ERK1/2, HBA1/HBA2, hemoglobin, Retnla, and SAA; Figure 4); among them, ARG1, Retnla, and HBA1/HBA2 were common DEPs to both groups. Network analysis suggested that GBFXD regulates mitochondrial energy metabolism and corrects M2 factor expression. Moreover, it was predicted that IFNG is significantly activated after GBFXD treatment, which supports our previous experimental findings. Upstream regulatory factor prediction also suggested that GBFXD significantly inhibits RICTOR. Taken together, the two significant upstream predictions indicate that GBFXD inhibits M2 macrophages in our model.

#### Validation

**FIGURE 5:**
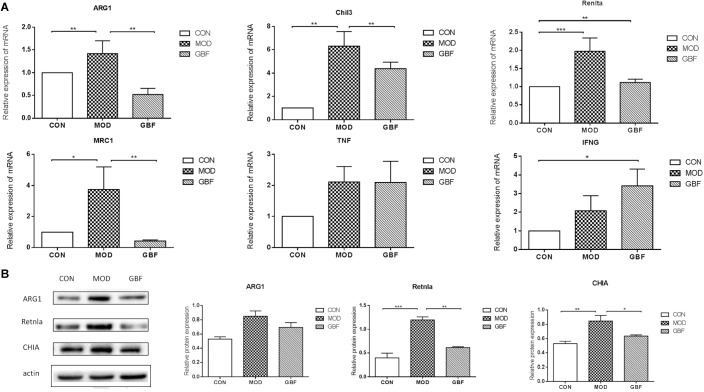
Validation of differentially expressed candidate proteins identified using proteomics. **(A)** qRT-PCR of six selected genes presented relative to control samples and normalized against GAPDH in each sample. **(B)** Western blot analysis shows decreased ARG1, Retnla, and CHIA levels in the GBFXD group (B). Values represent the means ± SE of three independent experiments with ≥3 replicates per experiment. ^∗∗^*p* < 0.01; ^∗∗∗^*p* < 0.0001; ^∗∗∗∗^*p* < 0.0001.

We further performed western blot analysis and found that expression levels of ARG1, CHIA, and Retnla in lung tissue were significantly decreased in the GBFXD group (Figure 5, Supplementary Data Sheet S1). This result is in agreement with the protein levels measured by iTRAQ analysis and the changing trends in transcription indicated by qRT-PCR. Therefore, our proteomic analysis output was deemed reliable.

## Discussion

Asthma is the most common chronic airway inflammation disorder whose incidence continues to increase worldwide. The pathophysiological features of asthma include continuous chronic airway inflammation, AHR, and airway remodeling. Moreover, there are dramatic epidemiological differences between childhood and adult asthma. As a first-line treatment for asthma, ICS is not well tolerated in children. Moreover, it may be difficult to diagnose asthma in certain children under the age of five (Pando et al., 2010; Klok et al., 2011; Bush, 2018; Ferrante and Grutta, 2018). As an alternative to ICS, several Asian countries employ GBFXD against asthma. Previous studies have shown that GBFXD reduces pulmonary inflammation during clinical remission in asthmatic mice (Huang et al., 2016; Lu et al., 2016) however, the mechanism remains unknown. Therefore, to understand the therapeutic role of GBFXD, we established a clinically consistent chronic asthmatic model and performed proteomic analysis. The study considerated the epidemiological characteristics of asthma in children; this subpopulation often presents with persistent allergen exposure inevitably (Szentpetery et al., 2017). Therefore, we increased the frequency of OVA challenges compared with that of our previous model. We compared both models and found that mice in the continuous OVA-induced model exhibit remarkable pathological features that were corroborated by lung function tests.

Alveolar macrophages, airway surface mucus, microorganisms, and airway epithelium constitute the first line of airway defense (Hussell and Bell, 2014). Macrophages can be adaptively polarized according to the metabolic characteristics of the tissue and immune environments. Macrophage polarization is categorized as the classical M1 type and the alternative M2 type. M1 polarization is pro-inflammatory and is initiated by adaptive immunity, whereas M2 polarization is anti-inflammatory and is critical for tissue damage and recovery during the late phases of infection (Wynn et al., 2013; Holtzman et al., 2014). M2 polarization mediates airway remodeling, matrix deposition, and other pathophysiological mechanisms in asthma (Wynn and Vannella, 2016). Th2 inflammatory factors are required to polarize macrophages toward the M2 phenotype (Dyken and Locksley, 2013). As OVA is often used to trigger Th2-type immune responses, the combination of OVA and RSV can aggravate Th2-type responses. Furthermore, excessive M2 macrophages are activated in lung tissue after RSV infection (Keegan et al., 2016; Naessens et al., 2016). Therefore, the increased M2 macrophage polarization exhibited by our model is credible.

Clinical studies have found that CD206^+^ macrophage is highly expressed in the trachea and bronchoalveolar lavage fluid in asthmatic patients. The degree of its expression is correlated with biological sex, asthma severity, and lung function. However, CD206^+^ macrophage may be insensitive to ICS treatment (Melgert et al., 2010, 2011; Draijer et al., 2016). Markers of alternatively activated macrophages include MRC1, ARG1, Retnla, and Chil3 and these are upregulated in the lungs. Herein, proteomic analysis indicated the presence of M2 macrophage markers, MRC1, ARG1, Retnla, Chil3, and CHIA in asthmatic. GBFXD treatment inhibited these factors (Supplementary Figure S1A). The inhibitory effect on M2 macrophages by GBFXD may result from its therapeutic effect in attenuating airway remodeling and ARH, which interact with each other and constitute the hidden pathogen inducing susceptibility to asthma. Therefore, GBFXD may represent an alternative therapeutic agent against asthma.

The upstream predictive function of our experiment indicated that IL-4/IL-5/IL-13 are significantly activated in this model. Arg1 is induced by IL-4/IL-13 in macrophages and counterbalances inflammatory signals. L-arginine metabolism in macrophages is a defining feature of alternative versus classical macrophage activation (Duan et al., 2011). Moreover, chitinase-like protein is elevated in serum and mainly in alveolar cells and macrophages of the lungs of asthmatic patients (Chupp et al., 2007). *ARG1* and *CHIL3* are well-recognized susceptibility genes closely associated with childhood asthma that increase AHR and weaken lung function; additionally, the polymorphic and epigenetic states of *ARG1* are associated with childhood asthma progression (Li et al., 2006; Ober et al., 2008; Salam et al., 2009; Cunningham et al., 2011). Retnla (resistin-like molecule-α), also known as FIZZ 1 (found in inflammatory zone 1), is also highly induced in allergic lung inflammation and bleomycin-induced lung fibrosis (Holcomb et al., 2000; Liu et al., 2014). Furthermore, macrophages can strongly express Retnla in chronic type 2 inflammation (Nair et al., 2003). In agreement, our proteomic analysis identified changes in these proteins that led to clinical outcomes. At the same time, lung function and pathology results also support these conclusions. Therefore, we believe that our model would be valuable in the study of chronic persistent asthma in children.

Mitochondria are the energy centers of cellular activity. They also maintain cellular ion homeostasis and lipid metabolism. Clinical studies have detected dysfunction and structural changes in the mitochondria of airway epithelial cells of asthmatic patients. Similar changes observed in asthmatic animal models indicated that abnormal oxidative stress, mitochondrial membrane potential, and energy metabolism occur (Mabalirajan et al., 2008; Aguilera and Bacsi, 2009; Zifa et al., 2012; Flaquer et al., 2014; Girodet et al., 2015; Wang et al., 2017). Th1/Th2 imbalance also adversely affects mitochondrial function (Chang et al., 2009; Pattnaik et al., 2016). Furthermore, arginine and chitinase metabolism affect mitochondrial oxidative stress (Zhang et al., 2009; Chao and Carter, 2015; Xu et al., 2016). In the present study, proteomic analysis showed that after GBFXD treatment, NDUFA1, NDUFA9, NDUFS7, ATP5F1, and HADH were upregulated while FIS1 was downregulated. Pathway and network analyses also suggested that GBFXD increases ATP synthesis and that mitochondrial complex 1 interacts with mitigating M2 macrophage markers (Supplementary Table S1 and Supplementary Figure S1B).

## Conclusion

Treatment with GBFXD can improve Th1/Th2 balance, inhibit alternatively activated macrophages, and reduce AHR, mucus secretion, and airway remodeling. We believe that GBFXD shows efficacy due to its mechanism of action, specifically the improvement in macrophage polarization and restoration of mitochondrial function.

## Ethics Statement

All experimental procedures were performed in accordance with the National Institutes of Health Guidelines for Laboratory Animals and approved by the Animal Ethics Committee of Nanjing University of Chinese Medicine (201810A026).

## Author Contributions

XZ conceived the project and designed the experiments. L-wL performed the data analysis and interpretation and Q-qX helped to write the manuscript. Q-qX, YL, MT, and Y-mD provided the experimental assistance. CD and YZ contributed to reagents, materials, and analysis tools.

## Conflict of Interest Statement

The authors declare that the research was conducted in the absence of any commercial or financial relationships that could be construed as a potential conflict of interest.

## Supplementary Material

FIGURE S1 Effect of GBFXD on expression of M2 and mitochondrial complex 1 marker in macrophages in mouse models. **(A)** Lung tissue was fixed and sections were processed for Immunofluorescence detection of M2 macrophage (CD206+f4/80+). **(B)** After macrophage collected from Balf, total mRNA was isolated and examined for transcript levels of the mitochondrial complex 1 markers (NDUFA1, NDUFA9, NDUFS7, ATP5F1) by RT-PCR analysis. Values represent the means ± SE of three independent experiments with 3 replicates per experiment. ^∗∗^*p* < 0.01; ^∗∗∗^*p* < 0.0001; ^∗∗∗∗^*p* < 0.0001.

TABLE S1 Primer sequences of *NDUFA1*, *NDUFA9*, *NDUFS7*, *ATP5F1*.
