# Supplementary figures and images for: Proteomic Analysis Provides Insights Into the Therapeutic Effect of GU-BEN-FANG-XIAO Decoction on a Persistent Asthmatic Mouse Model
Source: Front Pharmacol. 2019 May 7;10:441. doi: 10.3389/fphar.2019.00441 (PMC6514195; doi:10.3389/fphar.2019.00441)

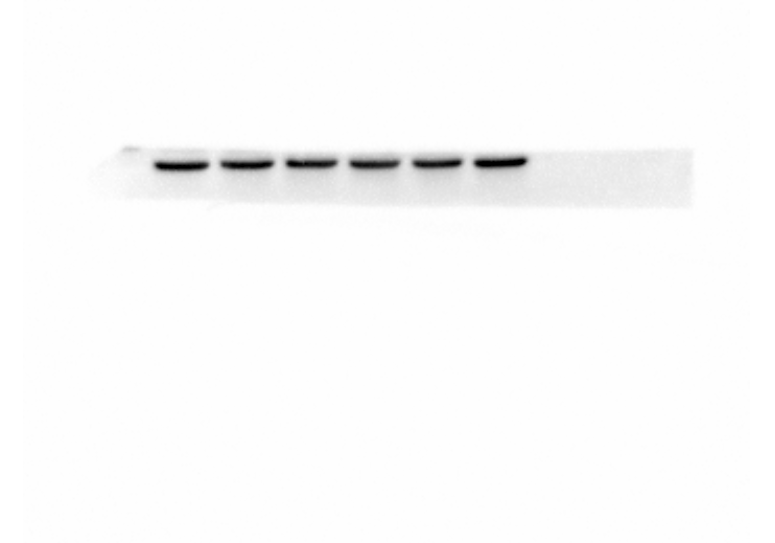

Supplement: FIGURE S1 — Effect of GBFXD on expression of M2 and mitochondrial complex 1 marker in macrophages in mouse models. (A) Lung tissue was fixed and sections were processed for Immunofluorescence detection of M2 macrophage (CD206+f4/80+). (B) After macrophage collected from Balf, total mRNA was isolated and examined for transcript levels of the mitochondrial complex 1 markers (NDUFA1, NDUFA9, NDUFS7, ATP5F1) by RT-PCR analysis. Values represent the means ± SE of three independent experiments with 3 replicates per experiment. ∗∗p < 0.01; ∗∗∗p < 0.0001; ∗∗∗∗p < 0.0001. [file Data_Sheet_1.ZIP › supplementary material/actin(CON-CRA MOD-CRA GBF-CRA CON-CPA MOD-CPA GBF-CPA).tif]

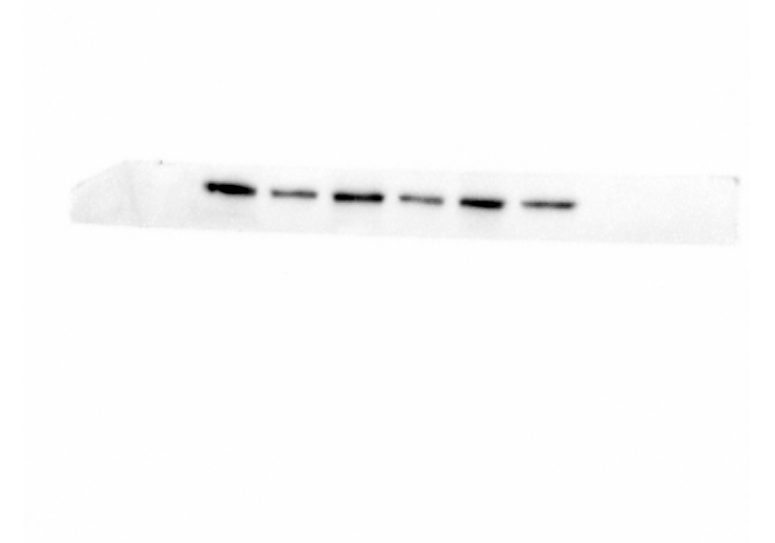

Supplement: FIGURE S1 — Effect of GBFXD on expression of M2 and mitochondrial complex 1 marker in macrophages in mouse models. (A) Lung tissue was fixed and sections were processed for Immunofluorescence detection of M2 macrophage (CD206+f4/80+). (B) After macrophage collected from Balf, total mRNA was isolated and examined for transcript levels of the mitochondrial complex 1 markers (NDUFA1, NDUFA9, NDUFS7, ATP5F1) by RT-PCR analysis. Values represent the means ± SE of three independent experiments with 3 replicates per experiment. ∗∗p < 0.01; ∗∗∗p < 0.0001; ∗∗∗∗p < 0.0001. [file Data_Sheet_1.ZIP › supplementary material/ARG1(CON-CRA MOD-CRA GBF-CRA CON-CPA MOD-CPA GBF-CPA).tif]

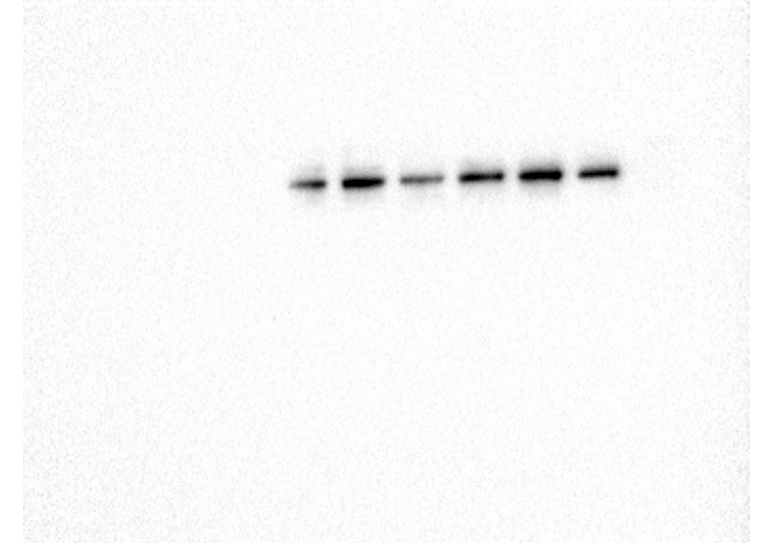

Supplement: FIGURE S1 — Effect of GBFXD on expression of M2 and mitochondrial complex 1 marker in macrophages in mouse models. (A) Lung tissue was fixed and sections were processed for Immunofluorescence detection of M2 macrophage (CD206+f4/80+). (B) After macrophage collected from Balf, total mRNA was isolated and examined for transcript levels of the mitochondrial complex 1 markers (NDUFA1, NDUFA9, NDUFS7, ATP5F1) by RT-PCR analysis. Values represent the means ± SE of three independent experiments with 3 replicates per experiment. ∗∗p < 0.01; ∗∗∗p < 0.0001; ∗∗∗∗p < 0.0001. [file Data_Sheet_1.ZIP › supplementary material/CHIA(CON-CRA MOD-CRA GBF-CRA CON-CPA MOD-CPA GBF-CPA).tif]

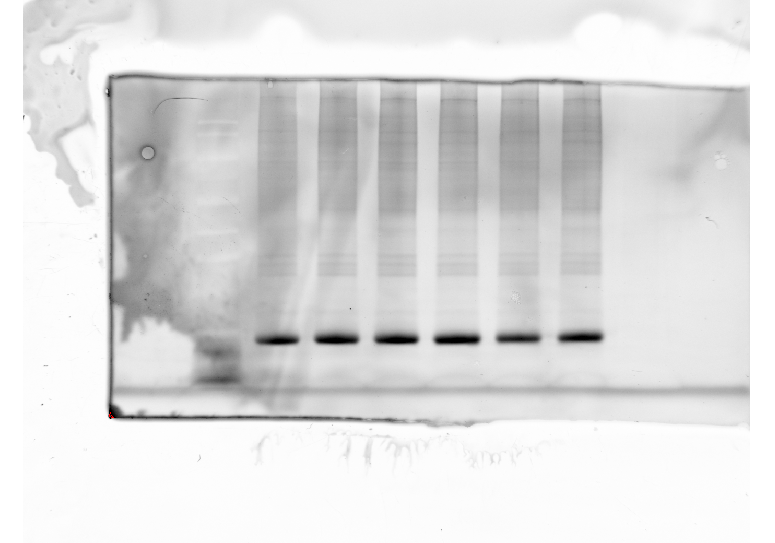

Supplement: FIGURE S1 — Effect of GBFXD on expression of M2 and mitochondrial complex 1 marker in macrophages in mouse models. (A) Lung tissue was fixed and sections were processed for Immunofluorescence detection of M2 macrophage (CD206+f4/80+). (B) After macrophage collected from Balf, total mRNA was isolated and examined for transcript levels of the mitochondrial complex 1 markers (NDUFA1, NDUFA9, NDUFS7, ATP5F1) by RT-PCR analysis. Values represent the means ± SE of three independent experiments with 3 replicates per experiment. ∗∗p < 0.01; ∗∗∗p < 0.0001; ∗∗∗∗p < 0.0001. [file Data_Sheet_1.ZIP › supplementary material/gel.tif]

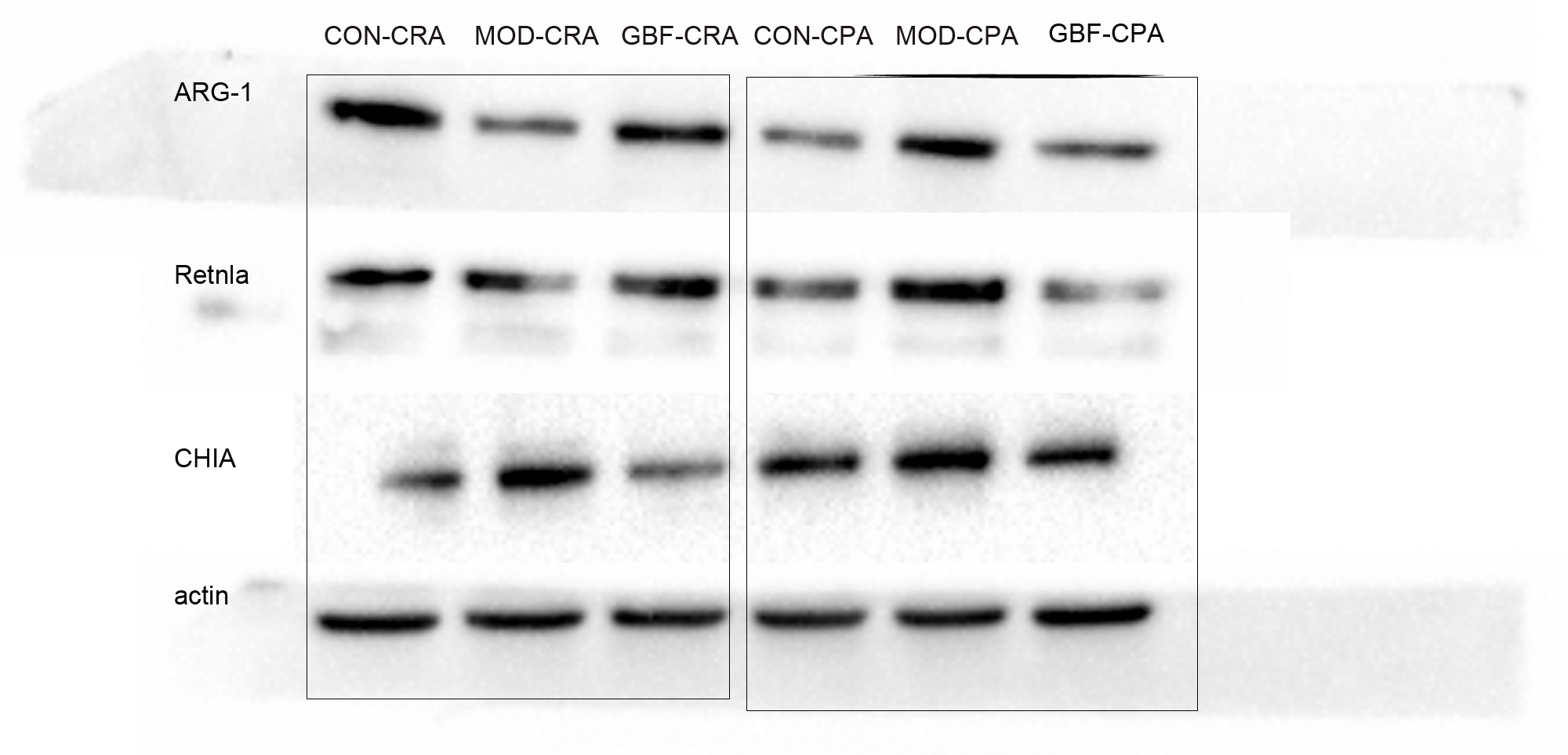

Supplement: FIGURE S1 — Effect of GBFXD on expression of M2 and mitochondrial complex 1 marker in macrophages in mouse models. (A) Lung tissue was fixed and sections were processed for Immunofluorescence detection of M2 macrophage (CD206+f4/80+). (B) After macrophage collected from Balf, total mRNA was isolated and examined for transcript levels of the mitochondrial complex 1 markers (NDUFA1, NDUFA9, NDUFS7, ATP5F1) by RT-PCR analysis. Values represent the means ± SE of three independent experiments with 3 replicates per experiment. ∗∗p < 0.01; ∗∗∗p < 0.0001; ∗∗∗∗p < 0.0001. [file Data_Sheet_1.ZIP › supplementary material/Grouping.tif]

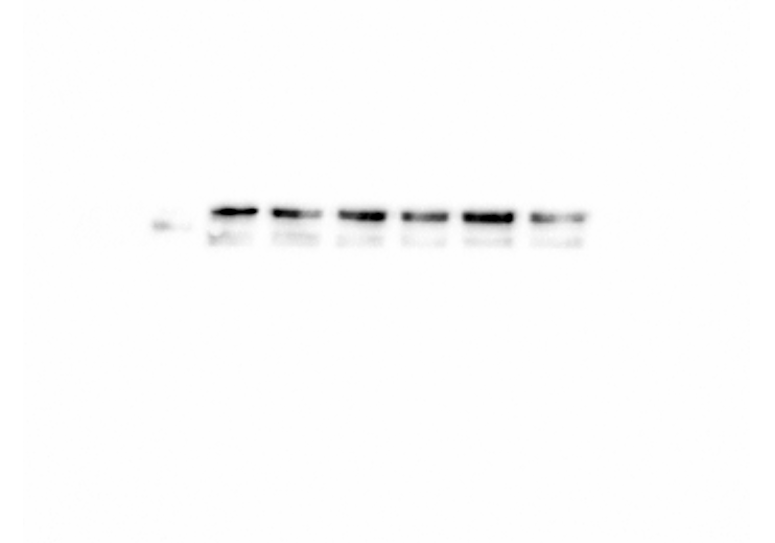

Supplement: FIGURE S1 — Effect of GBFXD on expression of M2 and mitochondrial complex 1 marker in macrophages in mouse models. (A) Lung tissue was fixed and sections were processed for Immunofluorescence detection of M2 macrophage (CD206+f4/80+). (B) After macrophage collected from Balf, total mRNA was isolated and examined for transcript levels of the mitochondrial complex 1 markers (NDUFA1, NDUFA9, NDUFS7, ATP5F1) by RT-PCR analysis. Values represent the means ± SE of three independent experiments with 3 replicates per experiment. ∗∗p < 0.01; ∗∗∗p < 0.0001; ∗∗∗∗p < 0.0001. [file Data_Sheet_1.ZIP › supplementary material/Rentla(CON-CRA MOD-CRA GBF-CRA CON-CPA MOD-CPA GBF-CPA).tif]

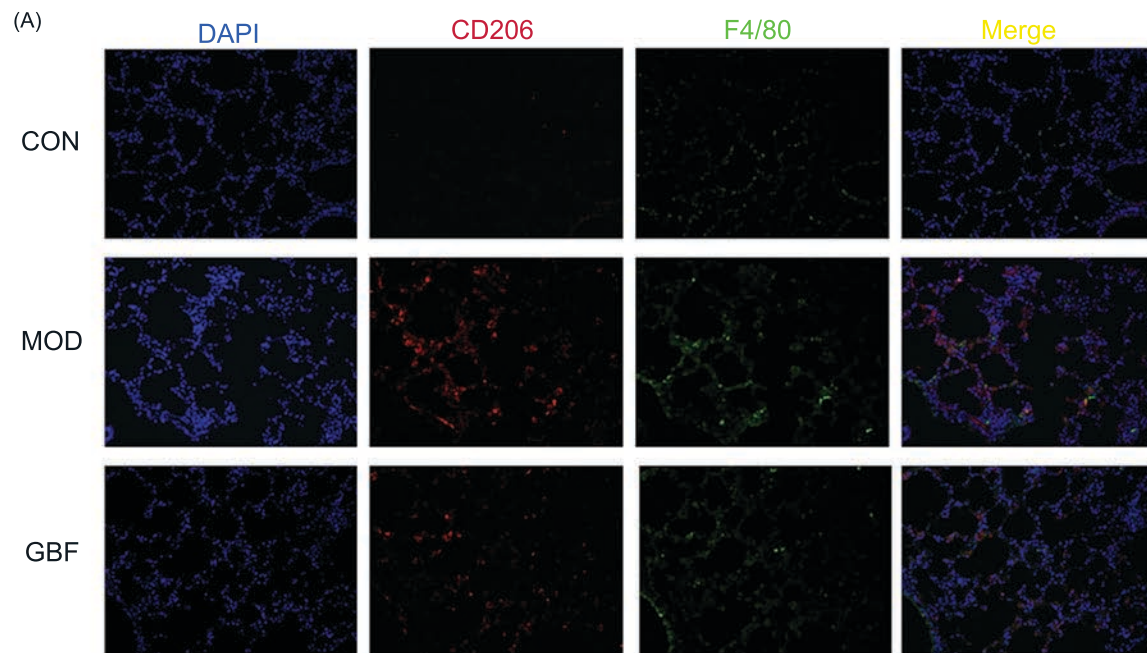

(B)

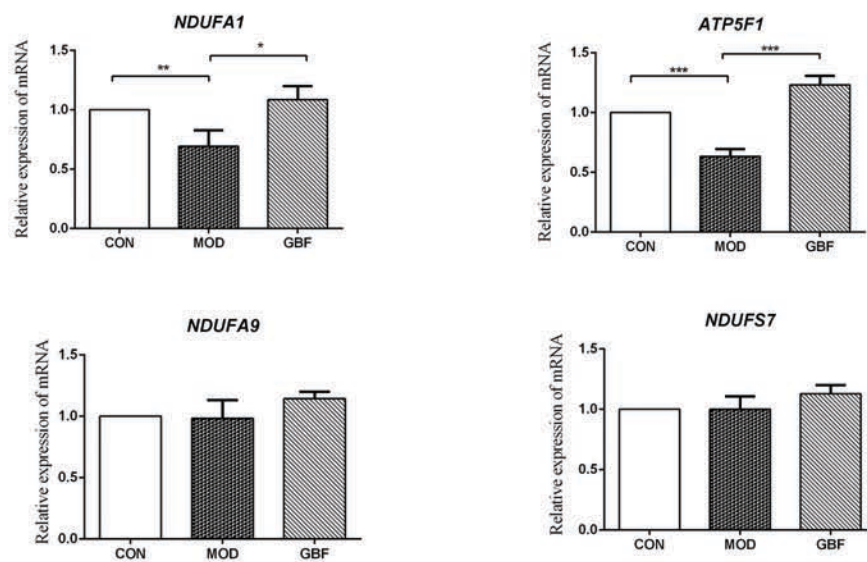

Supplement: TABLE S1 — Primer sequences of NDUFA1, NDUFA9, NDUFS7, ATP5F1. [file Data_Sheet_2.ZIP › Supplementary material-432211/supplementary Figure 1.pdf]
