# Supplementary material for: Proteomic Analysis Provides Insights Into the Therapeutic Effect of GU-BEN-FANG-XIAO Decoction on a Persistent Asthmatic Mouse Model
Source: Front Pharmacol. 2019 May 7;10:441. doi: 10.3389/fphar.2019.00441 (PMC6514195; doi:10.3389/fphar.2019.00441)
Supplement: TABLE S1 — Primer sequences of NDUFA1, NDUFA9, NDUFS7, ATP5F1. [file Data_Sheet_2.ZIP › Supplementary material-432211/supplementary Table 1.pdf]

Table 1 Primer sequences

Gene name	Forward primer	Reverse primer	Expected product size bp
Ndufa1	GGTGTCCACTGCGTACATCCAC	CGCGTTCCATCAGATACCACTGG	92
Ndufa9	ACGTGATGCCGACCGACCTG	GCCGTAGCACCTCAATGGACTTG	87
Ndufs7	CGCAGAGTTCATCAGAGTGTAGCC	CAGCCTTGGACACAGCAGACTG	91
Atp5f1	GGCACAGCAGGCACTGGTTC	TCCTTGCGACGCATCATGTTCTG	171
GAPDH	CGTGTTCCTACCCCCAATGT	TGTCATACTTGGCAGGTTT	104
